# Editorial: Salt tolerance in plants: molecular and functional adaptations

**DOI:** 10.3389/fpls.2023.1280788

**Published:** 2023-09-04

**Authors:** Ahmad Arzani, Suresh Kumar, Mohamed Magdy F. Mansour

**Affiliations:** ^1^ Department of Agronomy and Plant Breeding, College of Agriculture, Isfahan University of Technology, Isfahan, Iran; ^2^ Division of Biochemistry, ICAR-Indian Agricultural Research Institute, New Delhi, India; ^3^ Department of Botany, Faculty of Science, Ain Shams University, Cairo, Egypt

**Keywords:** abiotic stress, functional mechanisms., osmotic stress, oxidative stress, molecular, salinity, transcriptome

Salt stress is a significant obstacle to global food security and requires sustainable strategies to improve crop growth in harsh environments. Approximately one billion hectares of arable land worldwide were severely impacted by salinity in 2015, and it is expected that this issue will lead to a 50% decline by 2050 (Mansour and Hassan, 2022). Plants possess the ability to detect stimuli from their surroundings and regulate defense pathways through different regulatory networks in order to overcome abiotic stress. Salt tolerance is a complex trait that can be broken down into contributing factors and mechanisms. Over time and with increasing salt concentration, primary impairments occur in two phases: osmotic stress and specific ion toxicity. These can result in secondary stresses, such as oxidative stress and nutritional disorders. The aforementioned effects lead to a decline in plant growth, which can be attributed to disruptions in metabolic and physiological functions. These include reduced ability to absorb water and nutrients, membrane dysfunction, and disturbances in vital processes such as photosynthesis, respiration, and protein synthesis (Arzani and Ashraf, 2016; Kumar et al., 2017; Ragaey et al., 2022).

To enhance salt tolerance in crop plants, it is crucial to gain a thorough comprehension of the molecular mechanisms at play. Various genes are involved in salt tolerance, encompassing those responsible for signaling, ion transport, transcription factors, phytohormones, and the elimination of reactive oxygen species (ROS). It is imperative to delve deeper into these molecular and functional mechanisms to effectively devise breeding strategies aimed at augmenting salt tolerance in plants (Arzani and Ashraf, 2016; Formentin et al., 2018).

This Research Topic aims to provide an interdisciplinary understanding of how plants use biochemical, physiological, and molecular genetics mechanisms to adapt to salt stress. The contributions included in this Research Topic provide new insights into the responses and adaptations of various crops to salt stress and other abiotic stresses. The studies are summarized in **Table 1**, which highlights the plants used, the focus of the research, and some of the data obtained. The contributions are organized in chronological order based on their publication date.

**Table 1:** The focus of the seven studies in this Research Topic.

Seven papers quotation	Plant		Research focus	Data obtained
	Common name	Latin name		
1. Yang et al.	hybrid poplar	*Populus tremula* × *Populus alba*	Application of biotic stress-inducible synthetic promoters in genetic engineering	Synthetic promoters can be used for versatile control of gene expression in transgenic poplar and are useful tools to engineer stress-resilient woody plants.
2. Zhang et al.	Asparagus	*Asparagus officinalis*	Transcriptomic and metabolomic analyses of salt stress tolerance	Contributed mechanisms: a) transporters involved in K^+^/Na^+^ and water homeostasis; b) hormone (IAA and ABA) signal transduction c) sugar and amino acid metabolism for energy supply and osmotic regulation
3. Haber et al.	tomato	*Solanum lycopersicum*	The study aimed to investigate the impact of circular soil biosolarization (CSBS) on alleviating salt and nitrogen deficiency stress. This was accomplished through analysis of physiological responses, metabolome profiles, and microbiome composition.	CSBS significantly alleviated the effects of abiotic stress conditions. The presence of *Mycoplana* and *Kaistobacter* genera was found to promote plant health in the face of abiotic stress conditions. The results of this study bolster the proposed hypothesis and offer insights into the interplay among CSBS, soil ecology, and crop physiology in the presence of abiotic stress.
4. Sabeem et al.	date palm	*Phoenix dactylifera*	The potential benefits of *Piriformospora indica* in improving salt tolerance were investigated in date palm through physiological and gene expression analyses.	Fungus colonization benefits: a) enhanced activity of antioxidant enzymes b) altered expression of salt tolerance HKT1;5 and SOS1 genes. This study also revealed some indications of the beneficial effects of *P. indica* on plant growth when experiencing salt stress.
5. Perveen et al.	mango	*Mangifera indica*	Transcriptome response to salt stress was done in mango.	This investigation is one of the first studies in which transcriptome analysis of mango is used. This work extends our understanding of the transcriptome response to salt stress with some of the identified biological processes and pathways likely to function in adapting cells and mango plants to salt stress.
6. Zhu et al.	cucumber	*Cucumis sativus*	First, S-adenosylmethionine decarboxylase (*SAMDC*) genes were structurally and functionally characterized; Second, the impact of *CsSAMDC3* in salt stress adaptation was assessed by overexpressing in tobacco	Among four identified genes (*CsSAMDC1-4*) *CsSAMDC3* was abundantly expressed in fruits and flowers. This study suggests that *CsSAMDC3* could be used as a potential candidate gene to improve salt tolerance by regulating polyamine and antioxidant metabolism.
7. Tasnim et al.	cultivated rice and wild rice	*Oryza sativa* and *Oryza coarctata*, respectively	Wild halophytic and cultivated rice were grown in close proximity under salt stress. Growth, gene expression, and endophytic bacteria were assessed.	Cultivating both wild and cultivated rice together can significantly mitigate salt damage.

Yang et al. demonstrated the efficacy of synthetic stress-responsive promoters in genetically modified poplar plants subjected to osmotic stress conditions caused by salt stress and water deficit. Promoters play a crucial role in regulating gene expression by controlling the timing, location, and intensity of gene expression (Brooks et al., 2023). Stress-inducible promoters are preferred for genetic engineering as constitutive promoters can impose additional metabolic load on plant cells. Zhang et al. combine transcriptome and metabolome to uncover the mechanisms of adaptation to salt stress in asparagus. Their findings reveal differentially expressed genes (DEGs) related to ion transport, plant hormone response, cell division, and growth. The DEGs associated with ion transport between salt-tolerant and salt-sensitive genotypes highlight the importance of regulated influx and efflux of Cl^-^ and Na^+^ ions across organelle and cell membranes for salt tolerance. Likewise, the pictures emerging from their DEGs concerning the involvement of phytohormones in asparagus salt tolerance provide additional support for the hypothesis (Gharaghanipor et al., 2022) that hormone signal transduction pathways are a competitive alternative to other signaling pathways (calcium signaling pathways and MAPK signaling pathways) in modulating salt-stress responses.

Three papers focus on plant defense responses modulated by interactions with microbial communities and discuss strategies to enhance plant salt tolerance. In tomato, Haber et al. demonstrate a complex interaction between the soil microbiome of circular soil biosolarization treatment and the metabolome under salt and nitrogen deficit stress. Bacterial genera *Mycoplana* and *Kaistobacter* were found to promote host defense against abiotic stress. Similarly, Sabeem et al. show that the endophytic fungus *Piriformospora indica* alleviates salt stress in date palms by enhancing enzymatic antioxidant activities and modulating the expression of genes involved in Na^+^ and K^+^ ion channels in roots like *HKT1;5* and *SOS1*. Due to its mycorrhizal association, this fungus can colonize a wide range of plant species. It helps plants withstand environmental stresses such as drought, salinity, and nutrient-poor soils by improving their nutrient acquisition and water absorption capabilities. Tasnim et al conducted a co-growth experiment between cultivated rice plants and halophytic wild rice plants to examine the potential benefit of the halophyte on the survival of rice plants in a saline environment at the seedling stage. They also evaluated the endophytic microbial community in the roots and conducted transcriptome analysis of both cultivated and wild rice under both control and saline conditions in the pairing experiment. Overall, it was observed that the expression of transporters, carbohydrate metabolism, and photosynthesis was significantly heightened in cultivated rice when it was exposed to 100 mM NaCl stress alongside wild halophytic rice. This research highlights the fact that wild rice not only possesses salt-tolerant genes for its survival but also plays a vital role in bolstering the defense against salt stress in sensitive plants that inhabit the same area. These advantageous effects are achieved through diverse interactions between the wild rice and neighboring plants, which include the involvement of endophytic bacteria associated with wild rice.

Zhu et al. focused their research on S-adenosylmethionine decarboxylase (SAMDC), a crucial enzyme involved in the production of polyamines. They characterized four members of the cucumber *SAMDC* gene family (named *CsSAMDC1-4*) within the cucumber genome. The expression profile of these genes in roots and leaves was tested when exposed to salt stress. One specific member, *CsSAMDC3*, was subjected to further exploration by testing its expression patterns in various tissues and by overexpressing in tobacco plants to observe its response to salt stress. The results showed that *CsSAMDC3* could be used as a potential candidate gene to improve the salt tolerance of cucumber by regulating polyamine and antioxidant metabolism.

Finally, one contribution pertains to the transcriptome analysis aimed at gaining insights into the molecular response of mango to salt stress. Perveen et al. identified DEGs in salt-tolerant and sensitive mango genotypes, encompassing transcription factors, signal transduction, carbohydrate and energy metabolism, phytohormone biosynthesis and signaling, calcium signaling pathway, and protein kinases.

## Author contributions

AA: Writing – original draft. SK: Writing – review & editing. MMFM: Writing – review & editing.
